# Morbidity, mortality and mid-term follow-up in patients developing renal insufficiency after on-pump and off-pump coronary surgery

**DOI:** 10.1186/1749-8090-8-S1-O211

**Published:** 2013-09-11

**Authors:** K Ergunes, L Yilik, B Lafcı, E Celik, S Yazman, U Yetkin, T Goktogan, A Gurbuz

**Affiliations:** 1 Izmir Katip Celebi University Ataturk Training and Research Hospital, Department of Cardiovascular Surgery, Izmir, Turkey

## Background

We aimed to investigate factors affecting morbidity, mortality and survival in patients developing renal insufficiency after on-pump and off-pump coronary surgery.

## Methods

Between January 2002 and December 2009, 2034 patients underwent isolated on-pump and off-pump coronary revascularization in our clinic. Sixty-five patients were having postoperative renal insufficiency. The incidence of postoperative renal insufficiency determined as 3.4% (n=57) after on-pump and 2.1% (n=8) off-pump coronary revascularization.

## Results

Age, diabetes, preoperative renal insufficiency, and prolonged cardiopulmonary bypass time (CPB) were the independent predictive factors of postoperative renal insufficiency in patients undergoing on-pump coronary revascularization. Postoperative mortality rate was 12.5% (no=1) and 47.4% (n=27) in patients with postoperative renal insufficiency undergoing off-pump and on-pump coronary revascularization, respectively (P=0.124). Mean follow-up was 47.00±23.08 months and 44.97±20.96 months in patients with postoperative renal insufficiency undergoing off-pump and on-pump coronary revascularization, respectively. In follow-up, mortality rate was 37.5% (no=3) and 12.5% (n=7) in patients with postoperative renal insufficiency undergoing off-pump and on-pump coronary revascularization, respectively (P=0.098). Diabetes and reoperation for bleeding were the independent predictive factors of survival in patients with postoperative renal insufficiency undergoing on-pump coronary revascularization.

## Conclusions

Length of ICU stay and postoperative mortality rate were not significant statistically in patients with postoperative renal insufficiency undergoing off-pump and on-pump coronary revascularization. In follow-up, mortality rate was not significant statistically in patients with postoperative renal insufficiency undergoing off-pump and on-pump coronary revascularization.

